# Dietary quality linkage to overall competence at school and emotional disturbance in representative Taiwanese young adolescents: dependence on gender, parental characteristics and personal behaviors

**DOI:** 10.1186/s12937-018-0333-2

**Published:** 2018-02-22

**Authors:** Lin-Yuan Huang, Mark L. Wahlqvist, Meei-Shyuan Lee, Po-Huang Chiang

**Affiliations:** 1 Division of Preventive Medicine and Health Services Research, Institute of Population Health Sciences, National Health Research Institutes, 35 Keyan Road, Zhunan Town, Miaoli County 350 Taiwan, Republic of China; 2 School of Public Health, National Defense Medical Center, No.161, Sec. 6, Minchuan East Road, Taipei, 114 Taiwan, Republic of China; 3 Monash Asia Institute, Monash University, 5th Floor, H Building, 900 Dandenong Road, Caulfield East, Melbourne, VIC 3145 Australia; 4 College of Public Health, China Medical University, Taichung, Taiwan, Republic of China

**Keywords:** Gender, Parental characteristics, Personal behaviors, Dietary quality, Puberty, Junior high school, School performance

## Abstract

**Background:**

Child school performance during puberty may be at increased risk through emotional disturbance. It is hypothesized that this may be mitigated by dietary quality.

**Methods:**

In a nationally representative sample (Nutrition and Health Survey in Taiwan, NAHSIT), 1371 Taiwanese aged 11–16 years, overall competence at school, (OCS) and emotional status have been assessed by teachers with the SAED (Scale for Assessing Emotional Disturbance). Parents provided family socio-demographics and students completed a behavioral and dietary questionnaire (Youth Healthy Eating Index - Taiwan, YHEI-TW). Associations between emotional disturbance (ED), OCS and dietary quality (YHEI-TW) were assessed in multiple linear regression models with adjustments for covariates including parental characteristics, personal behaviors, body fatness and puberty.

**Results:**

Boys or girls with ED had a less favorable OCS (*p* < 0.001), minimally dependent on YHEI-TW. On multivariable analysis there was a more positive association between OCS and YHEI-TW among boys (β = 0.05, *p* < 0.01) and girls (β = 0.07, *p* < 0.001). Poor dietary quality was associated with ED, especially in girls (β =  − 0.06, *p* < 0.001). Additionally, parental characteristics, body fatness, and personal behaviors are associated with OCS. Puberty is associated with ED and may be indirectly linked to OCS.

**Conclusions:**

Unsatisfactory food intake is associated with the link between emotional disturbance and impaired school performance, as assessed by OCS, especially among girls. For both genders, socio-economic and behavioral factors including parenteral income, reading, screen viewing and smoking are modulators of this association. Puberty was a modifying factor in girls. Dietary quality is a relevant factor for health (ED) as well as education (OCS) during early adolescence.

**Electronic supplementary material:**

The online version of this article (10.1186/s12937-018-0333-2) contains supplementary material, which is available to authorized users.

## Background

Adolescence, nowadays considered the transitional period between childhood and adulthood, is a critical time in human development when pubertal change is underway, personal identity is being formatted and when future livelihood prospects are being established [1], especially through the education system. Minimizing emotional disturbance (ED) [2, 3] and optimizing school performance [4] at this time is a desirable research and policy objective. However, associations between ED and school performance are poorly documented [5, 6]. There is a growing recognition that items in the SAED (scale for assessing emotional disturbance) methodology [7] may be linked to overall competence at school (OCS) as a surrogate for school performance [8, 9] or learning disability [10]. A number of factors could affect any association between ED and OCS. These include home environment, school environment [11, 12], personal behaviors such as recreation and physical activity, cigarette smoking & alcohol usage [11], dietary quality [8, 13] nutritional status insofar as body fatness is concerned [14], and pubertal development [11].

*Diet* may also be a key factor in the expression of emotional disturbance [13, 14] and in cognitive and school performance [8, 15–18]. It may operate through energy regulation in conjunction with physical activity and sedentariness given that cognitive function appears amenable to measures which alter cellular energy regulation [19, 20], particularly in relation to the increasing burden of the metabolic syndrome and diabetes [21, 22]. Dietary quality has also been shown to play a role in emotion, mood [23] and mental performance [13] where limited food biodiversity, notably of plant foods, and degree of processing, with more ready-to-eat low nutrient density food items are the riskier consumption patterns [18, 23, 24]. However, there are limited observations about the connectedness of dietary patterns and brain function in relation to the child-adult transition. Partly, this is because of the biological heterogeneity and its secular trends at this time of reproductive transition.

*Parents* have the potential to affect both ED and OCS in many ways which reflect their continuing, if declining, role as providers of care, support and resources during their offspring’s adolescence [25]. This is likely to include pathways like diet [24, 26]. This is the case during elementary school where, particularly in girls vulnerable through low birth weight, nutritious diets have been associated with less likelihood of both emotional disturbance and poor school performance [13]. Further documentation and understanding of these phenomena may prompt guardians and mentors of young people to identify and act preventatively in regard to them, especially in regard to food choice and diet [2, 27].

*Our principal hypothesis* is that emotional disturbance (ED) during the child-to-adult transition is adversely associated with school performance as represented by overall competence (OC) and that dietary quality may mitigate such a linkage This association may, nevertheless, be modulated by various factors. The opportunity has been taken to study emotional disturbance and school performance among junior high school boys and girls in Taiwan in regard to diet and puberty, taking into account parental and various personal behavioral characteristics.

## Methods

### Study participants

Participants were adolescents aged 11–16 years (grade 7–9) who participated in the Nutrition and Health Survey in Taiwan (NAHSIT) 2010–2011. The original study is a national representative cross-sectional survey. All of Taiwan’s 358 townships/districts were classified into 5 strata (northern 1, northern 2, central, southern and eastern area) by geographical location and population density. The survey used the PPS (probabilities proportional to sizes) sampling method to select 1620 students from 30 junior high schools (6 schools from each stratum) randomly. NAHSIT comprises questionnaires administered by face-to-face interview of parents and students (including a food frequency questionnaire, 24-h dietary recall, pubertal development, personal behaviors from students; and family socio-demographics from parents). Physical examination (including body composition, blood pressure) was conducted and plasma metabolic analytes obtained though venipuncture by a health care professional. Thus, the questionnaire covered both dietary and non-dietary health factors [28].

The present study excluded participants who did not complete the emotional disturbance questionnaire, leaving 1371 (656 boys and 715 girls) junior high school students eligible for analysis. The survey was approved by the Institutional Review Board of the National Health Research Institutes, Taiwan.

### Measures

#### The scale for assessing emotional disturbance (SAED)

The modified Scale for Assessing Emotional Disturbance (SAED) was used to assess the NAHSIT students’ school and social performance, the original questionnaires being developed by Epstein and Cullinan [13, 29, 30]. The SAED scales for students were assessed by their teachers using the SAED questionnaire. The SAED is a rating scale designed to help identify students with emotional and/or behavioral difficulties at school. Its test-retest reliability coefficient is above > 0.80, and most of its subscales have inter-rater reliability coefficients over 0.79 [7]. The Chinese modification of SAED as used in this study has a high overall reliability of 0.92 and validity of 0.76 [29]. The SAED consist of a total of seven subscales including OC considered in this study as OCS and six other subscales. These are: Inability to Learn (IL, 8 items), Relationship Problems (RP, 6 items), Inappropriate Behavior (IB, 10 items), Unhappiness or Depression (UD, 7 items), Physical Symptoms or Fears (PF, 8 items)), Social Maladjustment (SM, 6 items), and Overall Competence at school (OCS, 7 items). OCS has been treated separately from SAED in this study (see below). Within the above subscales, items were scored by each student’s Class Mentor teacher. As previously described, the first six subscales (52 items) were scored on a four-point scale (0 = not a problem, 1 = mild problem, 2 = considerable problem, 3 = severe problem), and five-point scale for OCS (0 = far below average, 1 = below average, 2 = average, 3 = above average, and 4 = far above average). The raw scores for the SAED subscales were summed and converted into standardized scores (mean = 10 and SD = 3). Compared with the Taiwanese Non-Emotional Disturbance Norms, a substantially deviant SAED score (except OCS) is indicated by a score that above the 91 percentile (>90th percentile), which corresponds to Z-scores ≥13 [29]. Therefore, a score of 13 was chosen as the cut point. Children with a Z-score ≥ 13 were considered to have emotional disturbance (ED). IL, RP, IB, UD, and PF scores could be combined to produce a total score of ED characteristics. These five subscale scores plus SM score can be combined to yield an “SAED total” score [30, 31].

#### Overall competence at school (OCS)

OCS was used to assess the students’ overall performance and adaptation at school. The questions for OCS included (1) intellectual functioning, (2) family support for school, (3) overall level of academic functioning, (4) motivation for schoolwork, (5) level of peer support, (6) personal hygiene (e.g. grooming, dressing), and (7) interest in activities outside of school. Students with an OCS Z-score less than or equal to 6 (fell below the 9th percentile) were considered to have an unfavorable overall school performance [29, 30].

#### Dietary quality (YHEI-TW)

The Youth Healthy Eating Index-Taiwan (YHEI-TW) is a diet quality scoring system modified from the U.S. YHEI, which captures the adolescent’s diet quality by assessing his or her adherence to dietary guidelines [32]. The YHEI***–***TW scores were obtained from daily consumption of 11 components derived from food frequency questionnaires and a 24–hour dietary recall employed during NAHSIT: (1) whole grains (0 to ≥ 2 servings, 0–10 points), (2) vegetables (0 to ≥ 3 servings, 0–10 points), (3) fruits (0 to ≥ 3 servings, 0–10 points), (4) dairy (0 to ≥ 3 servings, 0–10 points), (5) meat ratio (0 to ≥ 20, 10–0 points), (6) snack foods (i.e., salty snacks and snacks with added sugar, 0 to ≥ 3 servings, 10–0 points), (7) sweetened beverages (0 to ≥3 servings, 10–0 points), (8) multivitamins (never-daily, 5–0 points), (9) fried foods outside of home (never-daily, 0–5 points), (10) consumption of breakfast (never to > 5 times/week, 0–5 points) and (11) dinner with family (prepared by family member, 5 points). It does not take into account butter/margarine and visible animal fat, which were part of the original YHEI (US), since consumption of these items has been negligible in Taiwan. In all cases, except component 11 (dinner patterns), scores were derived in a proportionate manner. Thus, total scores range from 0 to 90, where higher scores indicate better dietary quality [13, 33, 34].

#### Covariate measurements

Covariates were derived from questionnaires, by anthropometry and from laboratory measurements of metabolic analytes in the NAHSIT survey. Those considered in the present study included mother’s education (lower than university, university and above), household income (0–30,000, 30,000–50,000, 50,000–80,000, > 80,000 NTD/month where US$1 = NTD 30), ever smoking (no, yes), read during weekdays (0–1, 1–3, ≥3 h/day), watch TV during weekdays (0–1, 1–3, ≥3 h/day), play computer games during weekdays (0–1, 1–3, ≥3 h/day), moderate or heavy physical activity (0–30, ≥30 min/day; in accordance with Taiwanese recommendations) [35], BMI (underweight, normal, overweight, obesity; (the Childhood Obesity Expert Panel of the Taiwanese Department of Health defines ‘obesity’ and ‘overweight’ (≥95th and ≥85th percentile value of body mass index (BMI), respectively) using age- and gender-specificity percentiles for BMI (each gender and year of age from 2 to 18 had its own cut-off point for overweight and for obesity) [36], and puberty (boys: beard growth (not yet, at first, in progress, completed); girls: menarche (yes, no)).

### Statistical analysis

Chi-square tests were used to assess the significant differences between categorical variables. We used t tests to assess differences in continuous variables. Categorical variables are presented as percentages (%), and continuous variables are presented as means (standard errors, SE). Multiple linear regression models (MLRs) were used to test associations between the key determinants (household, personal behaviors, puberty, dietary quality, body fatness) and outcome measures (ED and OCS); and in the adjustments for mother’s education, household income, ever smoking, reading during weekdays, watching TV during weekdays, playing computer games during weekdays, moderate or heavy physical activity, pubertal development and BMI. Full models were considered which included all relevant variables. The regression coefficients reported are from these models. Statistical significance was set at *p* < 0.05. Data were analyzed using SAS 9.3 for Windows, weighted by SUDAAN [37]. SUDAAN was also used to adjust for the study design effect of cluster sampling to obtain unbiased estimates of the standard errors.

**Fig. 1:**
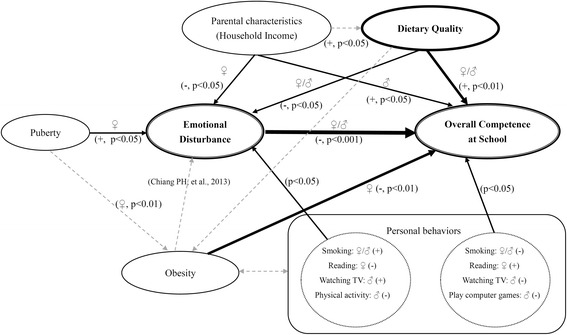
Pathways to emotional disturbance and overall competence at school in Taiwanese adolescents. Where an association is significant, the level of significance is indicated with the relevant *p* value and in accordance with gender. The findings represented in this figure are those of the present report except for “obesity→emotional disturbance” which have been reported in Reference [14] for Taiwanese schoolchildren

An overview of the study design and models is provided in Fig. 1.

## Results

### Overall competence at school

Table 1 shows the junior high school students’ characteristics by overall competence as an index of school performance. The mean age of the 1371 participants (51.7% are boys) was 13.6 years. There were 5.9% adolescents with unfavorable overall competence at school (OCS) (OCS Z-score ≤ 6). The percentage in boys was higher than girls (61.3% vs. 38.7%, *p* = 0.042). Adolescents had a better OCS when parents had a higher education. Adolescents in households where income was less than 30,000 NTD/month (about 1000 US dollars) had the most unfavorable OCS (34.5%), whereas this was only 11.2% in the > 80,000 NTD/month group (*p* < 0.001).

**Table 1:** Basic characteristics of junior high school students by Overall Competence (OCS) (*N* = 1371)

Characteristics^a^		OCS^b^		*P* value
	Overall	Z-score ≤ 6	Z-score > 6	
Participants (%)	1371 (100)	98 (5.9)	1273 (94.1)	
Mean age in years (SE)	13.6 (0.1)	13.3 (0.1)	13.6 (0.1)	0.130
Gender (%)				0.042
Boys	51.7	61.3	51.1	
Girls	48.3	38.7	48.9	
Father’s ethnicity (%)				0.777
Fukienese	76.6	71.9	76.9	
Hakka	13.0	15.8	12.8	
Mainlander	8.3	9.2	8.2	
Indigenes	2.2	3.2	2.1	
Father’s educational level (%)				0.008
Secondary education and below	40.5	61.9	39.1	
University and above	59.5	38.1	60.9	
Mother’s educational level (%)				0.018
Secondary education and below	33.4	47.5	32.5	
University and above	66.6	52.5	67.5	
Household income (%)				<0.001
0–30,000 NTD/month^c^	19.3	34.5	18.4	
30,000–50,000 NTD/month	21.7	24.1	21.6	
50,000–80,000 NTD/month	29.8	30.1	29.6	
> 80,000 NTD/month	29.2	11.2	30.3	
Ever smoking (%)				0.019
No	89.9	75.5	90.8	
Yes	10.1	24.5	9.19	
Drinking alcohol (%)				0.730
No	87.4	85.4	87.6	
Yes	12.6	14.6	12.4	
Read during weekdays (%)				0.010
0–1 (h/day)	45.6	67.2	44.3	
1–3	42.0	30.0	42.8	
≥ 3	12.4	2.9	13.0	
Watch TV during weekdays (%)				0.006
0–1 (h/day)	58.1	33.9	59.7	
1–3	33.7	53.2	32.5	
≥ 3	8.13	12.9	7.8	
Play computer games during weekdays (%)				0.064
0–1 (h/day)	62.3	48.8	63.2	
1–3	29.2	37.6	28.7	
≥ 3	8.45	13.6	8.1	
Moderate or heavy physical activity (%)				0.734
0–30 (min/day)	26.0	24.7	26.1	
≥ 30	74.0	75.3	73.9	

^a^Categorical variables are presented as percentage (%), and continuous variables are presented as mean (SE)

^b^Students with an OCS Z-score less than or equal to 6 were considered to have unfavorable overall school performance

^c^1 US dollars = 30 NTD

#### Personal Behaviors

Compared with favorable OCS, more smokers were found among unfavorable OCS (24.5%, *p* = 0.019), those who read only 0–1 h/day during weekdays (67.2%, *p* = 0.01), those who watched TV ≥3 h/day during weekdays (12.9%, *p* = 0.006), and those who played computer games ≥3 h/day during weekdays (13.6%, *p* = 0.064).

#### Dietary Quality

Participants with unfavorable OCS had lower dietary scores than did those with favorable OCS (44.0 ± 1.1 vs. 48.4 ± 0.5, *p* < 0.001) and also a higher consumption frequency (times/week) of fast foods (0.7 ± 0.1 vs. 0.4 ± 0.1, *p* = 0.029), sugary beverages (6.5 ± 0.6 vs. 5.3 ± 0.2, *p* = 0.053), and flavored milk (1.4 ± 0.3 vs. 0.7 ± 0.1, *p* = 0.015), but a lower cheese consumption (0.6 ± 0.1 vs. 0.9 ± 0.1, *p* = 0.035) (Table 2).

**Table 2:** Student food intakes, physical examination and metabolic analytes by Overall Competence (OCS)

		OCS^a^		*p* value
	Overall	Z-score ≤ 6	Z-score > 6	
Mean (SE) Dietary score (YHEI-TW)	48.1 (0.5)	44.0 (1.1)	48.4 (0.5)	<0.001
Mean (SE) Food consumption (times/week)				
Fast foods consumption	1.7 (0.1)	2.3 (0.3)	1.7 (0.1)	0.086
Fast foods from fast food chain store	0.5 (0.1)	0.7 (0.1)	0.4 (0.1)	0.029
Fast foods from non-fast food chain store	1.3 (0.1)	1.6 (0.3)	1.2 (0.1)	0.206
Sugary beverages consumption	5.4 (0.2)	6.5 (0.6)	5.3 (0.2)	0.053
Dairy products consumption				
Milk	3.5 (0.2)	3.0 (0.7)	3.6 (0.2)	0.499
Flavored milk	0.7 (0.1)	1.4 (0.3)	0.7 (0.1)	0.015
Yoghurt	0.5 (0.1)	0.7 (0.2)	0.5 (0.1)	0.342
Cheese	0.9 (0.1)	0.6 (0.1)	0.9 (0.1)	0.035
Development of puberty				
Menarche (girls only) (%)				0.929
Yes	94.0	93.5	94.0	
No	6.0	6.5	6.0	
Beard growth (boys only) (%)				0.036
Not yet	33.4	45.3	32.4	
At first	31.4	36.9	30.9	
In progress	33.8	13.2	35.4	
Completed	1.5	4.5	1.2	
Mean (SE) Body compositions				
Height (cm)	161 (0.3)	160 (1.2)	161 (0.3)	0.716
Weight (kg)	54.2 (0.5)	56.7 (1.7)	54.0 (0.5)	0.146
Body mass index (BMI, kg/m^2^)	20.8 (0.2)	21.9 (0.5)	20.8 (0.2)	0.046
Triceps skin fold thickness (TSF, mm)	16.6 (0.4)	17.2 (0.9)	16.6 (0.4)	0.496
Mid Arm Muscle Circumference (MAMC, cm)	20.0 (0.2)	21.5 (1.2)	19.9 (0.2)	0.221
waist circumference (WC, cm)	74.2 (0.5)	76.2 (1.2)	74.1 (0.5)	0.114
Mean (SE) Blood pressure (mmHg)				
Systolic blood pressure (SBP)	105 (0.5)	106 (1.5)	105 (0.5)	0.520
Diastolic blood pressure (DBP)	60.5 (0.7)	61.5 (1.2)	60.4 (0.7)	0.301
Mean (SE) Plasma metabolic analytes				
Fasting glucose (mg/dL)	95.4 (0.4)	95.2 (0.9)	95.4 (0.4)	0.835
Total cholesterol (mg/dL)	158 (1.4)	155 (3.3)	159 (1.4)	0.191
Triglycerides (mg/dL)	71.4 (1.8)	78.3 (5.5)	71.0 (1.7)	0.150
HDL cholesterol (mg/dL)	55.3 (0.7)	50.8 (1.9)	55.6 (0.7)	0.022
LDL cholesterol (mg/dL)	88.8 (1.2)	87.9 (3.2)	88.8 (1.2)	0.761
Uric acid (mg/dL)	5.8 (0.1)	6.2 (0.2)	5.74 (0.1)	0.063

^a^Students with an OCS Z-score less than or equal to 6 were considered to have unfavorable overall school performance

YHEI-TW, Youth Healthy Eating Index-Taiwan

#### Puberty

In regard to pubertal development, boys with ‘completed’ beard growth had the most unfavorable OCS (4.5%, *p* = 0.036) (Table 2). The means of YHEI-TW scores were not significant difference among onset of puberty in the present study (data not shown).

#### Body fatness and Metabolic analytes

Adolescents with an unfavorable OCS had a higher BMI and a lower HDL cholesterol than did those with a better OCS (BMI: 21.9 ± 0.5 vs. 20.8 ± 0.2, *p* = 0.046; HDL: 50.8 ± 1.9 vs. 55.6 ± 0.7, *p* = 0.022) (Table 2).

### Emotional disturbance

**Fig. 2:**
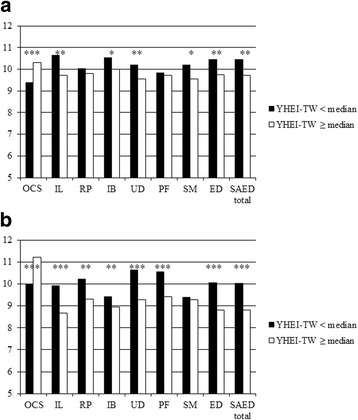
Gender-specific distribution of SAED Z-scores by YHEI-TW group. The median YHEI-TW for boys was 48.2, and 48.5 for girls. SAED, the Scale for Assessing Emotional Disturbance; OCS, Overall Competence at School; IL, Inability to Learn; RP, Relationship Problems; IB, Inappropriate Behavior; UD, Unhappiness or Depression; PF, Physical symptoms or Fears; SM, Socially Maladjusted; ED, Emotional Disturbance. * *p* < 0.05; ** *p* < 0.01; *** *p* < 0.001. **a** Boys SAED Z-score. **b** Girls SAED Z-score

Figure 2 shows the SAED Z-scores of adolescents by dietary quality (< or ≥ YHEI-TW median). The median YHEI-TW for boys was 48.2, and 48.5 for girls. Whether boys or girls, those with better diet quality had higher overall competence at school (*p* < 0.001). Boys with poorer diet quality had higher IL, IB, UD, SM, ED and total SAED Z-scores compared to those with better diet quality (*p* < 0.05). Among girls, those with poorer diet quality demonstrated more emotional disturbance (except SM) compared to those with better diet quality (*p* < 0.01).

### Emotional disturbance and overall competence at school

**Fig. 3:**
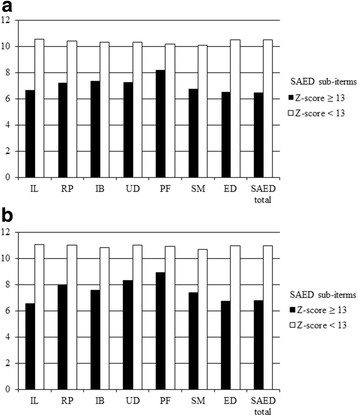
Z-scores of Overall Competence at school (OCS) by SAED sub-items and gender. Students with Z-scores ≥13 in any subscale were classified as having emotional disturbance. The t-test was used to compared the difference between two SAED sub-items groups; all of the results were significant (*p* < 0.001). **a** Boys OCS Z-score. **b** Girls OCS Z-score

Figure 3 shows the significant difference between total SAED and its sub-types with OCS as Z-scores < or ≥13. Whether boys or girls, students with emotional disturbance (Z-score ≥ 13) had significantly lower OCS Z-scores. Students without emotional disturbance had a better overall competence at school than those with emotional disturbance (*p* < 0.001). There were significant inverse associations between OCS and SAED and its sub-items among boys and girls (Additional file 1). When these relationships for SAED itself and its sub-items were adjusted for dietary quality, the β coefficients in the range of − 0.32 to − 0.85 were marginally reduced by the order of 0.01–0.05 in boys and girls, but remained significant; this indicated that a contribution from dietary quality to ED-linked OCS was small (Additional file 1).

### Predictive models for OCS

**Fig. 4:**
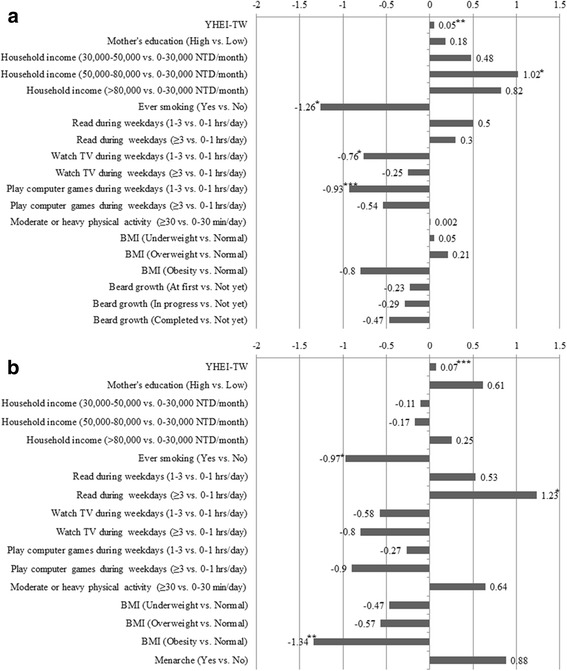
Socio-demographic, behavioral, nutritional and pubertal β-coefficients from MLRs^a^ for the Overall Competence Z-score by gender. ^a^ MLRs, Multiple Linear Regressions. The R^2^ for the complete model to predict OCS was 0.21 for boys and 0.22 for girls. * *p* < 0.05; ** *p* < 0.01; *** *p* < 0.001. **a** Boys. **b** Girls

The multiple linear regression analysis for OCS Z-scores, with reference to YHEI-TW and relevant co-variates, is shown in Fig. 4. In conjunction with other co-variates, there was a significant positive association between OCS and YHEI-TW among boys (β = 0.05, *p* < 0.01) and girls (β = 0.07, *p* < 0.001). Boys with a household income of 50–80,000 NTD/month had an OCS Z-score which was 1.02 greater than for those with < 30,000 NTD/ month (*p* < 0.05). Compared with not smoking, not watching TV, and not playing computer games 0–1 h/day during weekdays, boys who did had lower OCS Z-scores (β = − 1.26 (*p* < 0.05), − 0.76 (*p* < 0.05), and − 0.93 (*p* < 0.001) respectively. For girls, ever smoking and obesity were associated with lower OCS Z-scores than their non-smoking and normal weight counterparts (β was − 0.97 (p < 0.05) and − 1.34 (*p* < 0.01) respectively). Girls who read ≥3 h/day during weekdays had higher OCS Z-scores than did those who read 0–1 h/day (β = 1.23, *p* < 0.05).

The R^2^ for the complete model to predict OCS was 0.21 for boys and 0.22 for girls, explaining 21% and 22% of the variance respectively; without YHEI-TW in the model, R^2^ was 0.18 and 0.16 (18% and 16% of the variance respectively) (Fig. 4).

### Predictive models for SAED

Tables 3 and 4 show the multiple linear regressions for SAED subscales with reference to YHEI-TW and relevant co-variates. There was a significant inverse association between IL and YHEI-TW (β = − 0.05, *p* < 0.01) among boys. In the multivariable models, boys who had ever smoked had higher IL (β = 1.87, *p* < 0.05), IB (β = 1.88, *p* < 0.01), SM (β = 2.15, *p* < 0.05), ED (β = 1.65, *p* < 0.05), and SAED total (β = 1.73, *p* < 0.05) Z-scores. Compared with 0–1 h/day, boys who watched TV ≥ 3 h/day during weekdays were positively associated with PF (β = 2.03, *p* < 0.01) and ED (β = 1.53, *p* < 0.05) Z-scores, but negatively associated with the SM (β = − 0.99, *p* < 0.05) Z-score. Boys who engaged in moderate or heavy physical activity (≥ 30 min/day) had lower PF Z-scores than did those who reported 0–30 min/day (β = − 0.75, *p* < 0.05). Among girls, there were significant inverse associations between SAED subscale (except IB and SM) and YHEI-TW Z-scores. Girls’ households with 50,000–80,000 NTD/month income had lower IB, ED, and SAED total Z-scores (β values were − 0.69 (*p* < 0.05), − 0.97 (*p* < 0.01), and − 0.95 (*p* < 0.01), respectively) compared with those from the lowest household income group (0–30,000 NTD/month). Girls who had ever smoked had greater IL (β = 1.62, *p* < 0.01), IB (β = 1.96, *p* < 0.01), ED (β = 1.78, *p* < 0.05), and SAED total (β = 1.78, *p* < 0.05) Z-scores than those who never smoked. Unlike boys, there were significant negative associations between reading and IL, IB, SM, ED, and SAED total Z-scores among girls. In addition, girls who had entered menarche had higher PF (β = 1.2, *p* < 0.01) and SM (β = 0.34, *p* < 0.05) Z-scores than those who had not.

**Table 3:** Socio-demographic, behavioral, nutritional and pubertal β-coefficients from MLRs^a^ with R-square for SAED sub-items^b^ in boys

Characteristics	SAED Z-score							
	IL	RP	IB	UD	PF	SM	ED	SAED total
YHEI-TW	−0.05**	− 0.02	− 0.01	− 0.01	0.0002	− 0.02	− 0.03	− 0.03
Mother’s education (Ref: Low)								
High (University and above)	− 0.36	0.45	0.25	0.01	−0.08	0.25	−0.002	0.01
Household income (Ref: 0–30,000 NTD/month)^c^								
30,000–50,000 NTD/month	− 0.56	− 0.40	− 0.26	0.56	0.35	0.51	− 0.21	− 0.16
50,000–80,000 NTD/month	− 0.92	− 0.78	− 1.13	− 0.30	− 0.06	− 0.28	− 0.91	− 0.89
> 80,000 NTD/month	− 0.67	− 0.37	− 0.55	− 0.15	0.36	− 0.53	− 0.47	− 0.48
Ever smoking (Ref: No)								
Yes	1.87*	0.22	1.88**	1.23	0.38	2.15*	1.65*	1.73*
Read during weekdays (Ref: 0–1 h/day)								
1–3 h/day	− 0.26	−0.24	− 0.59	− 0.50	− 0.12	− 0.03	−0.44	− 0.42
≥ 3 h/day	− 0.22	−0.45	− 0.49	−0.30	0.05	0.11	−0.36	−0.34
Watch TV during weekdays (Ref: 0–1 h/day)								
1–3 h/day	0.31	0.21	−0.40	0.45	0.05	−0.38	0.14	0.11
≥ 3 h/day	1.19	0.50	1.02	1.61	2.03**	−0.99*	1.53*	1.40
Play computer games during weekdays (Ref: 0–1 h/day)								
1–3 h/day	0.46	0.26	0.72	0.66	0.49	0.55	0.65	0.66
≥ 3 h/day	0.002	0.41	0.78	0.08	−0.71	−0.35	0.20	0.17
Moderate or heavy physical activity (Ref: 0–30 min/day)								
≥ 30 min/day	0.07	0.35	−0.40	0.08	−0.75*	−0.37	− 0.14	−0.16
BMI (Ref: Normal weight)								
Underweight	0.08	0.10	−0.19	−0.22	− 0.35	0.35	− 0.11	−0.08
Overweight	−0.15	1.32	0.66	0.46	0.02	0.74	0.46	0.49
Obesity	0.88	0.50	−0.22	−0.06	−0.15	− 0.38	0.34	0.30
Beard growth (Boys only) (Ref: Not yet)								
At first	−0.39	0.004	−0.09	0.07	0.04	−0.03	−0.16	− 0.16
In progress	−0.62	− 0.74	−0.63	− 0.31	−0.43	− 0.34	−0.70	− 0.70
Completed	−0.02	− 0.50	0.55	− 0.70	−0.13	− 0.16	−0.09	− 0.10
R^2^ with YHEI-TW	0.175	0.049	0.124	0.097	0.083	0.123	0.133	0.134
R^2^ without YHEI-TW	0.159	0.047	0.124	0.096	0.083	0.119	0.128	0.129

^a^MLRs, Multiple Linear Regressions

^b^The Scale for Assessing Emotional Disturbance (SAED) sub-items including Inability to learn (IL), Relationship problems (RP), Inappropriate behavior (IB), Unhappiness or depression (UD), Physical symptoms or fears (PF), Socially maladjusted (SM), and Emotional Disturbance (ED)

^c^1 US dollars = 30 NTD

* *p* < 0.05; ** *p* < 0.01; *** *p* < 0.001

**Table 4:** Socio-demographic, behavioral, nutritional and pubertal β-coefficients from MLRs^a^ with R-square for SAED sub-items^b^ in girls

Characteristics	SAED Z-score							
	IL	RP	IB	UD	PF	SM	ED	SAED total
YHEI-TW	−0.05***	−0.05***	−0.03	− 0.07***	−0.06**	− 0.004	−0.06***	− 0.06***
Mother’s education (Ref: Low)								
High (University and above)	−0.19	0.14	0.45	0.15	0.88	0.17	0.26	0.26
Household income (Ref: 0–30,000 NTD/month)^c^								
30,000–50,000 NTD/month	0.12	−0.51	−0.41	−0.66	− 0.91	0.06	− 0.44	−0.42
50,000–80,000 NTD/month	−0.59	− 0.92	−0.69*	− 0.91	−1.09	−0.33	− 0.97**	−0.95**
> 80,000 NTD/month	0.01	0.02	−0.27	−0.35	− 0.59	−0.17	− 0.24	−0.24
Ever smoking (Ref: No)								
Yes	1.62**	0.45	1.96**	1.38	1.13	1.16	1.78*	1.78*
Read during weekdays (Ref: 0–1 h/day)								
1–3 h/day	−0.32	0.36	− 0.39	0.34	− 0.12	−0.07	− 0.13	−0.13
≥ 3 h/day	−1.35**	−0.44	− 0.73*	−0.17	− 0.20	−0.38*	− 0.91*	−0.90*
Watch TV during weekdays (Ref: 0–1 h/day)								
1–3 h/day	0.19	−0.38	0.25	−0.17	− 0.40	0.16	− 0.02	−0.01
≥ 3 h/day	0.33	−0.08	−0.20	0.01	−0.56	0.04	−0.03	− 0.03
Play computer games during weekdays (Ref: 0–1 h/day)								
1–3 h/day	0.21	0.10	0.02	0.12	0.13	−0.16	0.15	0.14
≥ 3 h/day	0.96	1.19	1.66	0.92	0.81	0.10	1.40	1.35
Moderate or heavy physical activity (Ref: 0–30 min/day)								
≥ 30 min/day	−0.49	−0.54	−0.84	0.18	−0.49	− 0.46	−0.59	− 0.60
BMI (Ref: Normal weight)								
Underweight	0.61	−0.31	0.24	0.61	0.32	0.33	0.43	0.44
Overweight	0.78	0.44	0.15	0.68	−0.14	0.27	0.54	0.54
Obesity	0.38	1.21	0.43	0.62	0.34	0.01	0.67	0.65
Menarche (Girls only) (Ref: No)								
Yes	−0.23	0.50	0.63	0.85	1.20**	0.34*	0.55	0.55
R^2^ with YHEI-TW	0.207	0.122	0.173	0.152	0.143	0.079	0.207	0.206
R^2^ without YHEI-TW	0.167	0.094	0.160	0.092	0.091	0.078	0.150	0.150

^a^MLRs, Multiple Linear Regressions

^b^The Scale for Assessing Emotional Disturbance (SAED) sub-items including Inability to learn (IL), Relationship problems (RP), Inappropriate behavior (IB), Unhappiness or depression (UD), Physical symptoms or fears (PF), Socially maladjusted (SM), and Emotional Disturbance (ED)

^c^1 US dollars = 30 NTD

* *p* < 0.05; ** *p* < 0.01; *** *p* < 0.001

The R^2^ for the complete model to predict SAED was 0.13 for boys and 0.21 for girls, explaining 13.4% and 20.6% of the variance respectively; without YHEI-TW in the model, R^2^ was 0.13 and 0.15 (12.9% and 15.0% of the variance).

Dietary quality (YHEI-TW) is associated with overall competence at school (OCS) as shown in Figs. 1 and 4 (β = 0.05, *p* < 0.01 for boys; 0.07 with *p* < 0.001 for girls). It is also associated with ED in boys and girls. In particular, there is a significant inverse association between IL and YHEI-TW (β = − 0.05, *p* < 0.01) among boys (Table 3). Among girls, there are significant inverse associations between the SAED subscales (except IB and SM) and YHEI-TW Z-scores (β for IL = − 0.05 (*p* < 0.001), for RP β = − 0.05 (*p* < 0.001), for UD β = − 0.07 (*p* < 0.001), for PF β = − 0.06 (p < 0.01), for ED β = − 0.06 (*p* < 0.001), and for SAED β = − 0.06 (*p* < 0.001)) (Fig. 1 and Table 4).

Thus, dietary quality appears to be linked to OCS directly and also indirectly via ED. The possibility that it might have been linked indirectly through an association with obesity was not evident in the present study (regression Model for BMI on YHEI-TW, β = 0.004 for boys and − 0.002 for girls, *p* > 0.05 (data not shown)).

In turn, dietary quality may have many determinants of the food system and choice. In this study, we only report household income as a surrogate for socio-economic status. It was positively correlated with YHEI-TW for boys and girls respectively (Fig. 1). Compared with those from lowest income group (0–30,000 NTD/month), students’ household income over 80,000 NTD/month had higher YHEI-TW scores (β = 5.45 (*p* < 0.001) for boys, and 3.19 (*p* < 0.05) for girls) (data not shown).

## Discussion

Unsatisfactory food intake is associated with the link between emotional disturbance and impaired school performance, as assessed by OCS, especially among girls. For both genders, socio-economic and behavioral factors including parenteral income, reading, screen viewing, and smoking are modulators of this association. Puberty was a modifying factor in girls. Dietary quality is a relevant factor for health (ED) as well as educational (OCS) during early adolescence.

### Emotional disturbance and overall competence at school (OCS)

For the various measures of emotional disturbance reflected in SAED, there were consistently negative associations with OCS. These findings confirm several published studies [38–41]. Even so, a number of factors may be contributory to this relationship. The focus of the present investigation is how dietary quality might affect any such linkage. Linear regression for this association for boys and girls explains 44% and 42% of the variance, respectively (data not shown). There is a small contribution of dietary quality to OCS via ED evident in the β coefficients of the MLRs which are uniformly less when these models are adjusted for YHEI-TW However, dietary quality in boys and girls is directly associated with OCS in our analysis. This link remains independent of other covariates on MLRs. Thus OCS is dependent on ED, YHEI-TW and various independent covariates, namely, in boys, household income (favorably, screen viewing (unfavorably)) along with smoking (unfavorably) and, in girls, weekday reading (favorably) and body fatness (unfavorably along with smoking (unfavorably)).

### Dietary quality and pattern

The present study has found that better dietary quality is associated with better overall competence at school and lower emotional disturbance. This supports reports, like that among Australian adolescents, that ‘Western’ dietary patterns characterized by energy-dense take-away ‘fast’ foods, plentiful red and processed meat, soft drinks, and other deep-fried and refined foods scores are associated with poorer academic performance (especially in mathematics and reading) [42]. For 14-year olds, this dietary pattern is associated with diminished cognitive performance 3 years later, at age 17 [43]. On the other hand, fruit and vegetable intake has a positive association with school performance among adolescents, as found in the Palestinian Gaza Strip [44]. Insofar as dairy foods are concerned, there may be differences within the category since OCS was less with flavored milk and greater with cheese; since all dairy products were treated together in the YHEI-TW, this may have accounted for non-significant associations on multiple linear regression for dairy. There is evidence that vitamin K-2 as found in cheese may play a role in brain function [45–47]. Of particular interest, regular breakfast, in its own right, is associated with better school performance, suggesting a role for diurnal dietary pattern in brain function [8, 17].

Previous studies in Taiwan show a dose-response for food diversity scores and health outcomes in adults, but comparable data are not available for children [48]. There is a gradient across the range, so that the upper quartile probably constitutes a desirable goal for food diversity at all ages. UN system data for dietary diversity and household food security support this position [49, 50].

The present study adds to a growing literature on the utility of YHEI-TW in children of dominantly Chinese ancestry and culture in the evaluation of diet in child development. These include studies of birth weight, food patterns and school performance [13] and of intergenerational dietary interplay in communities [34].

### Parental characteristics

In this study, adolescents who had a better OC in school had parents with higher educational achievements. Children’s school performance has previously been found to be strongly and positively associated with parental education [51].

Where household incomes were less, OCS was correspondingly less good. Similarly, in the US, youths with serious emotional disturbance have been observed to come from lower income families than for those youths without disturbance [52].

### Personal behaviors

Smoking, reading, watching TV, playing computer games, and limited physical activity in the present study were associated with less OCS and with emotional disturbance. Likewise, smoking among adolescents has been associated with poor school performance and less study time [53]. Although causality is difficult to ascertain, clustering of riskier behaviors among adolescents is found with mood disturbance and includes smoking, substance use and self-harm or suicidal attempts [54].

### Who are the students at risk-puberty and gender?

Although it is a difficult life stage to evaluate, because of its variable and changing time of onset, it is one of the most socio-biologically critical developmental periods. It is known that school environments in Taiwan, including their food systems and recreational settings, are associated with pubertal development, although differentially in girls and boys [11]. In the present study, the peri-pubertal period is evidently one of emotional and educational vulnerability, notably in girls, judged by both the indices of learning and mood examined. After the menarche, PF and SM were increased. However, dietary quality is a mitigating factor and food intake an addressable behavior.

### What are the factors of greatest concern for adolescents in development and schooling?

Of the potential independent risk factors for emotional disturbance or school performance (OCS or sub-scales), diet (YHEI-TW), socio-economic advantage (household income) and reading were favorable, while screen viewing, smoking, puberty and greater BMI were unfavorable. These findings closely correspond to a Korean study of health behaviors and academic performance in adolescents, especially for diet and smoking, except that it found physical activity to be significant [26]. In the present study, for girls, we found BMI to be independently associated with OCS, which may have captured physical activity information.

In an Australian study of children and adolescents 4–17 years of age, internet usage and electronic gaming was found to be a mental health risk [55]. There are likely to be different internet usage patterns in a dominantly Chinese as opposed to multicultural, but mainly European, Australian society with different cultural restraints and expectations of young adolescents. The gender difference in Taiwan of internet associations would also suggest this in the present study. It is an area in which more detailed analysis is required.

### Strengths and weaknesses

The adolescent population studied was representative of that in Taiwan, albeit dominantly Han Chinese with ancient and recent origins in mainland China. Indigenes were also studied and no differences in findings were apparent; this may represent a sampling limitation as previously observed, even though over-sampling of minorities, with SUDAAN adjustment for representativeness, has been undertaken in the present study [56]. Further cross-cultural extrapolation may be unwarranted, especially to other food cultures and educational systems. Other studies suggest limited generalizability to North-East Asian settings in general, but not dominantly European settings as in Australia, as discussed above. The cross-sectionality of the study begs the question of the medium to long-term health and performance outcomes of diet, along with other significant associated factors. As always with observational studies, residual confounding may be a problem in interpretation.

### Implications for health and nutrition policy and practice

Population-wide and representative evidence is provided in this study for children of dominantly Chinese ancestry and culture that dietary quality, along with parental input and personal behaviors is associated with emotional status and school performance. Intervention studies in support of these findings would add confidence to policy and practice which sought to enhance school performance by diet. In the meantime, household, school and community encouragement for healthier dietary patterns should be a low risk-high benefit option.

## Conclusions

The most supportable link of dietary quality to OCS is apparently direct rather than through ED to which it is also related in the present study. While ED is associated with OCS, and the intake of foods of limited nutritional value is seen with ED, the linkage of ED to OCS is minimally dependent on dietary quality. For both genders, socio-economic, parental education, reading or screen viewing, and smoking were associated with ED and OCS These factors may modulate the association between ED and OCS. Thus, the ways by which diet may affect OCS as a basis of school performance are likely to be complex.

## Additional file

Additional file 1: β-coefficients from the linear regressions for the Overall Competence (OC) and SAED Z-score. (DOCX 14 kb)

## Abbreviation

ED: Emotional disturbance
IB: Inappropriate behavior
IL: Inability to learn
NAHSIT: Nutrition and health survey in Taiwan
OC: Overall competence
OCS: Overall competence at school
PF: Physical symptoms or fears
RP: Relationship problems
SAED: Scale for assessing emotional disturbance
SM: Social maladjustment
UD: Unhappiness or depression
YHEI-TW: Youth healthy eating index – Taiwan

## References

[1] WHO. HEALTH FOR THE WORLD'S ADOLESCENTS: A second chance in the second decade. 2014. http://apps.who.int/adolescent/second-decade/section4/page1/Mental-health-issues.html. Accessed 12 May 2017.

[2] McGorry PD, Goldstone S (2011). Is this normal? Assessing mental health in young people. Aust Fam Physician.

[3] Rickwood DJ, Deane FP, Wilson CJ (2007). When and how do young people seek professional help for mental health problems?. Med J Aust.

[4] Serbin LA, Stack DM, Kingdon D (2013). Academic success across the transition from primary to secondary schooling among lower-income adolescents: understanding the effects of family resources and gender. J Youth Adolesc.

[5] Wiley AL, Siperstein GN, Forness SR, Brigham FJ (2010). School context and the problem behavior and social skills of students with emotional disturbance. J Child Fam Stud.

[6] Wagner MM (1995). Outcomes for youths with serious emotional disturbance in secondary school and early adulthood. Futur Child.

[7] Epstein MH, Cullinan D, Harniss M, Ryser G (1999). The scale for assessing emotional disturbance: test-retest and Interrater reliability. Behav Disord.

[8] Ho CY, Huang YC, Lo YT, Wahlqvist ML, Lee MS (2015). Breakfast is associated with the metabolic syndrome and school performance among Taiwanese children. Res Dev Disabil.

[9] Fu ML, Cheng L, Tu SH, Pan WH (2007). Association between unhealthful eating patterns and unfavorable overall school performance in children. J Am Diet Assoc.

[10] Lane KL, Carter EW, Pierson MR, Glaeser BC (2006). Academic, social, and behavioral characteristics of high school students with emotional disturbances or learning disabilities. J Emot Behav Disord.

[11] Chiang PH, Huang LY, Lee MS, Tsou HC, Wahlqvist ML (2017). Fitness and food environments around junior high schools in Taiwan and their association with body composition: gender differences for recreational, reading, food and beverage exposures. PLoS One.

[12] Chiang PH, Wahlqvist ML, Lee MS, Huang LY, Chen HH, Huang ST (2011). Fast-food outlets and walkability in school neighbourhoods predict fatness in boys and height in girls: a Taiwanese population study. Public Health Nutr.

[13] Lee MS, Huang LY, Chang YH, Huang ST, Yu HL, Wahlqvist ML (2012). Lower birth weight and diet in Taiwanese girls more than boys predicts learning impediments. Res Dev Disabil.

[14] Chiang PH, Huang LY, Lo YT, Lee MS, Wahlqvist ML (2013). Bidirectionality and gender differences in emotional disturbance associations with obesity among Taiwanese schoolchildren. Res Dev Disabil.

[15] Kim SY, Sim S, Park B, Kong IG, Kim JH, Choi HG (2016). Dietary habits are associated with school performance in adolescents. Medicine.

[16] Leventakou V, Roumeliotaki T, Sarri K, Koutra K, Kampouri M, Kyriklaki A (2016). Dietary patterns in early childhood and child cognitive and psychomotor development: the Rhea mother-child cohort study in Crete. Br J Nutr.

[17] Adolphus K, Lawton CL, Dye L (2013). The effects of breakfast on behavior and academic performance in children and adolescents. Front Hum Neurosci.

[18] Burrows T, Goldman S, Pursey K, Lim R (2017). Is there an association between dietary intake and academic achievement: a systematic review. J Hum Nutr Diet.

[19] Hardman RJ, Kennedy G, Macpherson H, Scholey AB, Pipingas A (2016). Adherence to a Mediterranean-style diet and effects on cognition in adults: a qualitative evaluation and systematic review of longitudinal and prospective trials. Front Nutr.

[20] Santomauro F, Lorini C, Tanini T, Indiani L, Lastrucci V, Comodo N, Bonaccorsi G (2014). Adherence to Mediterranean diet in a sample of Tuscan adolescents. Nutrition.

[21] Esposito K, Chiodini P, Maiorino MI, Bellastella G, Panagiotakos D, Giugliano D (2014). Which diet for prevention of type 2 diabetes? A meta-analysis of prospective studies. Endocrine.

[22] Lee AM, Gurka MJ, DeBoer MD (2016). Trends in metabolic syndrome severity and lifestyle factors among adolescents. Pediatrics.

[23] Isasi CR, Ostrovsky NW, Wills TA (2013). The association of emotion regulation with lifestyle behaviors in inner-city adolescents. Eat Behav.

[24] Burrows T, Goldman S, Olson RK, Byrne B, Coventry WL (2017). Associations between selected dietary behaviours and academic achievement: a study of Australian school aged children. Appetite.

[25] Farooq MS, Chaudhry AH, Shafiq M, Berhanu G (2011). Factors affecting students’ quality of academic performance: a case of secondary school level. JQTM.

[26] So ES, Park BM (2016). Health behaviors and academic performance among Korean adolescents. Asian Nurs Res (Korean Soc Nurs Sci).

[27] Undheim AM, Lydersen S, Kayed NS (2016). Do school teachers and primary contacts in residential youth care institutions recognize mental health problems in adolescents?. Child Adolesc Psychiatry Ment Health.

[28] Nutrition and Health Survey in Taiwan 2010 (2016). Health promotion administration.

[29] Cheng LY (2001). Scale for assessing emotional disturbance: Examiner's manual (Chinese edition).

[30] Epstein MH, Cullinan D (1998). Scale for assessing emotional disturbance: Examiner's manual.

[31] Epstein MH, Cullinan D, Ryser G, Pearson N (2002). Development of a scale to assess emotional disturbance. Behav Disord.

[32] Feskanich D, Rockett HR, Colditz GA (2004). Modifying the healthy eating index to assess diet quality in children and adolescents. J Am Diet Assoc.

[33] Yu XL (2007). Overall dietary quality indices and nutritional knowledge, attitude, and status in the children of Taiwan.

[34] Wahlqvist ML, Huang LY, Lee MS, Chiang PH, Chang YH, Tsao AP (2014). Dietary quality of elders and children is interdependent in Taiwanese communities: a NAHSIT mapping study. Ecol Food Nutr.

[35] Ku PW, Fox KR, McKenna J, Peng TL (2006). Prevalence of leisure-time physical activity in Taiwanese adults: results of four national surveys, 2000-2004. Prev Med.

[36] Chu NF, Pan WH (2007). Prevalence of obesity and its comorbidities among schoolchildren in Taiwan. Asia Pac J Clin Nutr.

[37] Shah BV, Barnwell BG, Bieler GS (2001). SUDDAN. User's manual. Research Triangle Park, NC: Research Triangle Institute.

[38] Mazzone L, Ducci F, Scoto MC, Passaniti E, D’Arrigo VG, Vitiello B (2007). The role of anxiety symptoms in school performance in a community sample of children and adolescents. BMC Public Health.

[39] Moksnes UK, Espnes GA, Haugan G (2013). Stress, sense of coherence and emotional symptoms in adolescents. Psychol Health.

[40] Vuontela V, Carlson S, Troberg AM, Fontell T, Simola P, Saarinen S, Aronen ET (2013). Working memory, attention, inhibition, and their relation to adaptive functioning and behavioral/emotional symptoms in school-aged children. Child Psychiatry Hum Dev.

[41] Wood JJ (2006). Effect of anxiety reduction on children's school performance and social adjustment. Dev Psychol.

[42] Nyaradi A, Li J, Hickling S, Foster JK, Jacques A, Ambrosini GL, Oddy WH (2015). A western dietary pattern is associated with poor academic performance in Australian adolescents. Nutrients.

[43] Nyaradi A, Foster JK, Hickling S, Li J, Ambrosini GL, Jacques A, Oddy WH (2014). Prospective associations between dietary patterns and cognitive performance during adolescence. J Child Psychol Psychiatry.

[44] Abudayya A, Shi Z, Abed Y, Holmboe-Ottesen G (2011). Diet, nutritional status and school performance among adolescents in Gaza strip. East Mediterr Health J.

[45] Ferland G (2012). Vitamin K, an emerging nutrient in brain function. Biofactors.

[46] Li J, Lin JC, Wang H, Peterson JW, Furie BC, Furie B (2003). Novel role of vitamin k in preventing oxidative injury to developing oligodendrocytes and neurons. J Neurosci.

[47] Walther B, Karl JP, Booth SL, Boyaval P (2013). Menaquinones, bacteria, and the food supply: the relevance of dairy and fermented food products to vitamin K requirements. Adv Nutr.

[48] Lee MS, Huang YC, Su HH, Lee MZ, Wahlqvist ML (2011). A simple food quality index predicts mortality in elderly Taiwanese. J Nutr Health Aging.

[49] Ruel MT (2003). Operationalizing dietary diversity: a review of measurement issues and research priorities. J Nutr.

[50] Hoddinott J, Yohannes Y (2002). Dietary diversity as a food security indicator. Food consumption and nutrition division discussion paper no. 136.

[51] Ruijsbroek A, Wijga AH, Gehring U, Kerkhof M, Droomers M (2015). School performance: a matter of health or socio-economic background? Findings from the PIAMA birth cohort study. PLoS One.

[52] Mark TL, Buck JA (2006). Characteristics of U.S. youths with serious emotional disturbance: data from the National Health Interview Survey. Psychiatr Serv.

[53] Yorulmaz F, Akturk Z, Dagdeviren N, Dalkilic A (2002). Smoking among adolescents: relation to school success, socioeconomic status nutrition and self-esteem. Swiss Med Wkly.

[54] Pan PY, Yeh CB (2015). Mood disturbance in adolescents screened by the mood disorder questionnaire predicts poorer social adjustment. J Adolesc Health.

[55] Rikkers W, Lawrence D, Hafekost J, Zubrick SR (2016). Internet use and electronic gaming by children and adolescents with emotional and behavioural problems in Australia - results from the second child and adolescent survey of mental health and wellbeing. BMC Public Health.

[56] Chiang PH, Wahlqvist ML, Huang LY, Chang YC (2013). Leisure time physical activities and dietary quality of the general and indigenous Taiwanese populations are associated with fat distribution and sarcopenia. Asia Pac J Clin Nutr.

