# Supplementary material for: Dietary quality linkage to overall competence at school and emotional disturbance in representative Taiwanese young adolescents: dependence on gender, parental characteristics and personal behaviors
Source: Nutr J. 2018 Feb 22;17:29. doi: 10.1186/s12937-018-0333-2 (PMC5822521; doi:10.1186/s12937-018-0333-2)
Supplement: Supplementary file 1 — β-coefficients from the linear regressions for the Overall Competence (OC) and SAED Z-score. (DOCX 14 kb) [file 12937_2018_333_MOESM1_ESM.docx]

**Additional file 1**

β-coefficients from the linear regressions for the Overall Competence (OC) and SAED Z-score

|  | OC Z-score | | | |
| --- | --- | --- | --- | --- |
|  | Boys | | Girls | |
| SAED sub-items | Crude model | Adjusted model^a^ | Crude model | Adjusted model^a^ |
| IL | -0.66*** | -0.65*** | -0.85*** | -0.83*** |
| RP | -0.48*** | -0.46*** | -0.51*** | -0.47*** |
| IB | -0.43*** | -0.39*** | -0.55*** | -0.52*** |
| UD | -0.47*** | -0.44*** | -0.49*** | -0.46*** |
| PF | -0.32*** | -0.29*** | -0.32*** | -0.27*** |
| SM | -0.34*** | -0.30*** | -0.51*** | -0.46*** |
| ED | -0.63*** | -0.60*** | -0.75*** | -0.73*** |
| SAED | -0.62*** | -0.60*** | -0.77*** | -0.74*** |

^a^ Model adjusted for dietary quality (YHEI-TW).

***p<0.001
